# Evaluation of Molar Buccolingual Inclination on Digital Models in Untreated Subjects with Near-Normal Occlusion

**DOI:** 10.3390/jcm15124514

**Published:** 2026-06-11

**Authors:** Leandra Garcia Jorge, Chenshuang Li, Jaime Guberman, Normand Boucher, Todd Welsh, Chun-Hsi Chung

**Affiliations:** Department of Orthodontics, University of Pennsylvania School of Dental Medicine, 240 S. 40th Street, Philadelphia, PA 19104-6303, USA; lpgarcia@upenn.edu (L.G.J.); lichens@upenn.edu (C.L.); jgub@upenn.edu (J.G.); nboucher@upenn.edu (N.B.); tmwelsh@upenn.edu (T.W.)

**Keywords:** buccolingual inclination, first molars, near-normal occlusion, 3D digital models, Dolphin Imaging, coronal section, CBCT

## Abstract

**Objectives:** To evaluate the buccolingual inclination of maxillary and mandibular first molars using 3D digital models in adolescents and adults with near-normal occlusion. **Material and Methods:** Forty-one untreated adults (mean: 41.7 years) and sixteen adolescents (mean: 15.2 years) with near-normal occlusion were selected. Each subject’s 3D digital models were imported into Dolphin Imaging software. The coronal cross-section was attained in no more than a 1.2 mm slice, using a section that best included both right and left first molars while both arches were in occlusion. The long axis of each molar was determined by drawing a line from the midpoint between the buccal and lingual cusp tips to the midpoint of the buccolingual width at the cervical region. The inclination angle of each first molar was then measured. **Results:** Adolescents and adults showed a similar directional trend in maxillary buccal inclination and mandibular lingual inclination of the first molars. The mean buccal inclination for the maxillary molar in adolescents was 9.0° and 9.1° in adults. The mean lingual inclination for the mandibular first molar in adolescents was 14.5° and 15.6° in adults. **Conclusions:** (1) In near-normal occlusion, measured from 3D digital models, maxillary first molars showed approximately 9° buccal inclination, and the mandibular first molars showed about 15° lingual inclination. (2) Within the limitations of the present sample and measurement protocol, 3D digital models provided reproducible crown-based measurements of first molar buccolingual inclination, with values similar to those previously reported in CBCT-based studies. Direct validation against CBCT measurements in the same subjects and evaluation in patients with transverse discrepancies or post-treatment records are needed before broader diagnostic or outcome-related claims can be made.

## 1. Introduction

Literature suggests that complete buccolingual uprighting of molars may not reflect natural dentofacial anatomy [1,2,3]. Wilson described the occlusal surfaces of posterior teeth as following a natural compensatory curve, present as concave in the mandible and convex in the maxilla, with the lower posterior teeth exhibiting a lingual inclination and the upper posterior teeth displaying a buccal inclination [4]. Dempster et al. found that in skulls with typical dentition, the maxillary posterior teeth inclined buccally, while the mandibular posterior teeth inclined lingually [5]. Establishing a proper curve of Wilson is essential for maintaining proper oral health, and alterations can lead to balancing interferences and impaired masticatory function [6,7,8].

This philosophy has informed generations of orthodontic diagnosis and treatment planning. The American Board of Orthodontics (ABO) assesses clinically acceptable buccolingual inclination of posterior teeth by evaluating the height differences between buccal and lingual cusps [9]. For quantitative assessment, the ABO utilizes a step gauge, requiring the lingual cusps of maxillary molars to be positioned more occlusally than the buccal cusps within 1 mm and the buccal cusps of mandibular molars to be positioned more occlusally than the lingual cusps within 1 mm. This reinforces the inclination of molars within a normal and physiologic range.

Of additional interest are the changes in buccolingual inclination throughout growth. Historically, plaster models have been used to assess these changes. For example, Marshall et al. observed that between the ages of 7.5 and 26.4 years, the maxillary first molars uprighted by 3.4°, while the second molars showed a greater uprighting of 6° [10]. In contrast, the mandibular first molars became more upright by 5.1°, and the second molars by 7.6° [10]. Sayania et al. also assessed longitudinal changes in buccolingual inclination from age 6 to 16, quantifying them in millimeters. They found that mandibular first molars initially erupted with a lingual crown inclination at age 6, gradually uprighted in a buccal direction as growth progressed, though a slight lingual inclination persisted by age 16. During this period (ages 6–16), maxillary molars in boys uprighted by approximately 0.4 mm, while mandibular molars uprighted by about 0.5 mm. Similarly, maxillary first molars erupted with a buccal crown inclination, became more upright lingually over time, yet they retained a mild buccal inclination at age 16. Among girls, maxillary molars uprighted by roughly 0.6 mm and mandibular molars by 0.3 mm between ages 6 and 16 [11]. In the recent literature, CBCT has been used to quantify the degree of buccolingual inclination and molar uprighting at various ages, yielding similar results [12,13]. For example, a CBCT analysis of the long axis of the teeth by Yang and Chung revealed that the maxillary first molar buccal inclination was 8.7° in adolescents and 4.7° in adults, while the mandibular first molar lingual inclination was 13.3° in adolescents and 13.1° in adults [12]. Collectively, these observations suggest that maintaining some degree of inclination—rather than eliminating it through aggressive uprighting—may better preserve functional arch relationships, enhance occlusal stability, and reduce the risk of relapse or iatrogenic effects such as balancing interferences.

More recent literature has begun to investigate posterior tooth inclinations in relation to the underlying skeletal bases in normal occlusion using CBCT. Lee et al. analyzed transverse dimensions and molar inclinations in 53 untreated adults and found that maxillary first molars showed a buccal inclination of 5.3° while mandibular first molars showed a lingual inclination of 14.4°. Furthermore, a correlation existed between the maxillomandibular skeletal differential and the inclination of the first molars [14]. In a study by Miner et al., 241 CBCT scans of untreated children with and without crossbite were analyzed to assess jaw widths and first molar inclinations [15]. As part of their findings, they reported that the non-crossbite group consisted of patients who had normal transverse relationships but also included patients with skeletal transverse discrepancy that was disguised by dental compensation. They concluded that patients without dental crossbites can have significant skeletal transverse discrepancies. This underscores the importance of thoroughly evaluating the buccolingual inclination of molars, as the presence or absence of dental compensations may indicate underlying transverse discrepancies.

CBCT represents a valuable tool for assessing buccolingual inclination in both clinical and research settings. Accurate evaluation of crown inclination is a critical component of orthodontic diagnosis, essential for achieving stable outcomes and a functional, healthy occlusion. However, CBCT is not typically included in routine orthodontic diagnostic records due to its high cost and associated radiation exposure. Its use is generally reserved for specific clinical indications, such as impacted teeth or skeletal asymmetries. Prescribing CBCT imaging without clear clinical justification raises ethical concerns, particularly when less invasive and more cost-effective alternatives are available. 3D Digital models have been widely adopted in modern orthodontic practice, allowing for a comprehensive evaluation of occlusion across multiple planes of space at various regions of the dental arches. This enables clinicians to conduct both qualitative and quantitative assessments with greater precision and flexibility. This raises an important question: can 3D digital models reliably be used to evaluate the buccolingual inclination of molars? To our knowledge, this question has not been answered in the literature, and there have been no reported normative values for molar inclinations using digital models on patients with near-normal occlusion. Therefore, the purpose of this study was to assess and provide norms for the buccolingual inclination of maxillary and mandibular first molars in adolescents and adults with near-normal occlusions using 3D digital models.

## 2. Materials and Methods

IRB approval was obtained from the University of Pennsylvania before data collection. 3D Digital models were created from intraoral scans acquired using iTero machines (Align Technology, Temple, AZ, USA) at two private orthodontic clinics in Pennsylvania. All images were oriented and evaluated using Dolphin Imaging 3D software (version 12.0.61; Dolphin Imaging and Management Solutions, Chatsworth, CA, USA).

Forty-one untreated adults (9 males and 32 females; mean age: 41.7 years) and sixteen adolescents (10 males and 6 females; mean age: 15.2 years) were selected. 3D Digital model records were collected for all subjects during the initial orthodontic evaluation, before sample selection, for confirmation of near-normal occlusion. The inclusion criteria were the following: (1) without previous orthodontic treatment; (2) less than 5 mm of crowding or spacing per arch; (3) fully erupted maxillary and mandibular first molars with complete root formation; (4) skeletal Class I (ANB 0–4°); (5) without missing teeth other than third molars; and (6) Angle’s Class I or near-Class I (within 2 mm) molar relationship. The exclusion criteria were: (1) prior orthodontic treatment; (2) posterior crossbite; (3) supernumerary teeth; (4) craniofacial deformities; and (5) periodontal disease.

Using Dolphin Imaging (version 12.0.61, Dolphin Imaging and Management Solutions, Chatsworth, CA, USA), the 3D digital models were oriented so that the occlusal plane was parallel to the floor. From the frontal view, canines, premolars, and molars were aligned on the same axial plane, with the palatal midline located on the mid-sagittal plane. The sagittal axis was defined at the buccal groove of the maxillary first molar. The coronal cross-section was obtained in no more than a 1.2 mm slice, using a section that best included both right and left first molars while both arches were in occlusion. The sagittal axis was first defined at the buccal groove of the right maxillary first molar. Once the coronal slice was obtained from the right maxillary first molar, the digital models were analyzed from their left side to ensure that the cut was also made on the left maxillary first molar.

**Figure 1:**
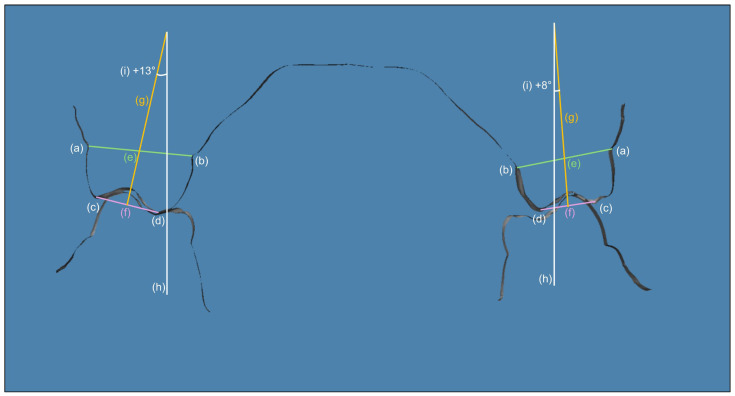
The inclination angle of the maxillary first molar is measured relative to a true vertical reference line. (a) buccal cervical point; (b) palatal cervical point; (c) buccal cusp tip; (d) palatal cusp tip; (e) cervical midpoint; (f) buccal and lingual cusp midpoint; (g) molar long axis; (h) true vertical line; (i) measured inclination angle. A negative (–) inclination angle was given when the crown tilted lingually and a positive (+) when it tilted buccally.

**Figure 2:**
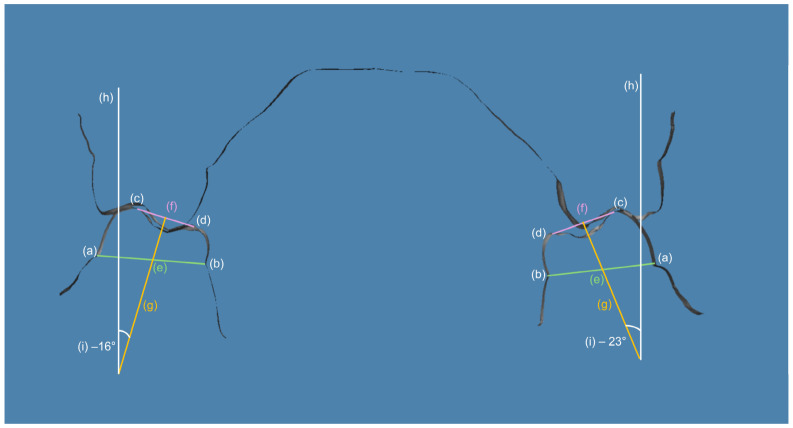
The inclination angle of the mandibular first molar is measured relative to a true vertical reference line. (a) Buccal cervical point; (b) lingual cervical point; (c) buccal cusp tip; (d) lingual cusp tip; (e) cervical midpoint; (f) buccal and lingual cusp midpoint; (g) molar long axis; (h) true vertical line; (i) measured inclination angle. A negative (–) inclination angle was given when the crown tilted lingually and a positive (+) when it tilted buccally.

The long axis of each molar was determined by drawing a line from the midpoint between the buccal and lingual cusp tips to the midpoint of the buccolingual width at the cervical region. The inclination angle of each maxillary and mandibular first molar was then measured relative to a true vertical reference line (Figure 1 and Figure 2). A negative (–) value was assigned when the crown tilted lingually in relation to the roots, and a positive (+) value was recorded when the crown inclined buccally.

All measurements and data were completed by the same examiner (L.G.J.) All data were tested for reliability and reproducibility by conducting intraexaminer trials. To test intraexaminer reproducibility, eight patient records were randomly selected, reoriented, measured, and remeasured three weeks after the test by the same examiner. A paired *t*-test was used to determine whether there was a significant difference between the 2 measurements taken at the 2 times. The intra-class correlation coefficient (ICC) of the measurements was also calculated by using the two-way random model to test the absolute agreement. The Shapiro–Wilk and Kolmogorov–Smirnov tests were used to assess the normal distribution for each side of each group. Descriptive statistics, including means, standard deviations, and ranges, were calculated for the measurements. To determine whether there were significant differences between the adolescent and adult sets of measurements, a Student’s *t*-test was performed. In addition, a mixed-effects model with patient as a random effect was used to avoid the cluster effects from using data from both left and right sides. And the results of single measurements were reported. The significance was determined at *p* < 0.05. A power analysis was conducted with an effective size of 0.8. With D = 1.0, α = 0.05, two tails, and β = 0.2, the sample size should be 17/group for the *t*-test. Except for the ICC, which was calculated by utilizing the IBM SPSS software (Statistical Package for Social Sciences version 26.0, Chicago, IL, USA), statistical analyses were performed using GraphPad Prism (Version 8.2.1, GraphPad Software, San Diego, CA, USA).

## 3. Results

### 3.1. ICC

The intra-examiner reliability test showed no significant differences between the original and repeated transverse measurements (*p* > 0.05). The Intraclass Correlation Coefficient (ICC) ranged from 0.94 to 0.99, indicating high reproducibility (Table 1).

### 3.2. Normality and Lognormality Tests

All groups tested passed both the Shapiro–Wilk and Kolmogorov–Smirnov tests for normality. There was consistent evidence that the data for all groups approximated a normal distribution, validating the use of parametric statistical methods for further analysis.

### 3.3. Buccolingual Maxillary Molar Inclination Measurements

Both groups exhibited positive measurements, indicating a similar directional trend in maxillary buccal inclination. The adult maxillary values exhibited a broad range (19.4°), with a mean of 9.1 ± 4.6°, indicating moderate variability. The adolescents’ maxillary values showed a slightly wider range (20.6°) than those of adults, with a comparable mean (9.0°). The adolescents’ maxillary values had greater variability than those of adults, as seen by the higher standard deviation of 5.2°. There was no statistically significant difference between maxillary values for adolescents and adults (*p* = 0.98 for *t*-test, and *p* = 0.73 for mixed-effects model). The slight mean difference (−0.02) with wide confidence intervals suggests a high degree of overlap between groups. Variances were not significantly different (*p* = 0.3), supporting the homogeneity of variance assumption (Table 2 and Table 3).

### 3.4. Buccolingual Mandibular Molar Inclination Measurements

Both groups exhibited negative measurements, indicating a similar directional trend in mandibular lingual inclination. The mean mandibular value for adults (−15.6°) was slightly higher than for adolescents (−14.5°), with higher variability, as indicated by the differences in the range of values (22.3° vs. 18.2°). The mean difference is 1.1°, which means that, on average, adolescents had slightly smaller negative measurements than adults. There was no statistically significant difference in mandibular molar inclinations between adolescents and adults (*p* = 0.3 for *t*-test, and *p* = 0.38 for mixed-effects model). The confidence interval included zero, reinforcing that the observed difference was not statistically significant and could be due to random variation. In conclusion, although the mean molar inclination in adolescents was slightly less negative than in adults, this difference was small, not statistically significant, and might be due to chance (Table 4 and Table 5).

A post hoc power analysis was conducted with the mixed-effects model, and the actual observed D is 0.0044 for the maxillary comparison and 0.227 for the mandibular comparison, indicating the current study found no difference between age groups because there likely is no meaningful effect, not because of low power.

## 4. Discussion

While the use of CBCT imaging to measure and establish norms for the buccolingual inclination of maxillary and mandibular molars is well-documented in the literature, 3D digital models offer a viable tool for these measurements. To the best of our knowledge, this is the first study using 3D digital models to establish a method to do the measurements and provide the norms on the buccolingual inclination of maxillary and mandibular first molars.

In our study, the long axis of the first maxillary and mandibular molars was defined as the line connecting the midpoint of the buccal and lingual cusp tips to the midpoint of the buccolingual width at the cervical base (near the furcation), adapting methods described by Alkhatib and Chung and Yang and Chung [1,12]. Alternatively, Kasai and Kawamura mapped the long axis on CBCT images by connecting the midpoint of the crown width to a point one-third of the distance from the root apex [16]. In contrast, Shewinvanakitkul et al. defined the long axis of the mandibular first molars as a line extending from the central groove to the midpoint of the root apices [17].

Across our dataset, the maxillary molars exhibited consistent and nearly identical buccal inclinations between the two groups evaluated, with mean values of 9.0° in adolescents and 9.1° in adults. Our findings did not demonstrate additional molar uprighting when comparing the adolescent and adult groups. Yet, the adolescent group had a slightly higher standard deviation, indicating greater variability within this group. In previous CBCT-based studies, Yang and Chung reported a mean maxillary first molar buccal inclination of 8.7° in adolescents and 4.7° in adults, and Alkhatib and Chung documented a buccal inclination of 4.9° for the maxillary first molars in adults [1,12].

In our findings, adolescents exhibited a lingual inclination of the mandibular molar (mean = −14.5°), similar to that of adults (mean = −15.6°). Interestingly, this was slightly less lingually inclined than in adults. This may be attributed not only to the small sample size (*n* = 16) but also to the older age distribution (mean age = 15.2 years) of our sample. In our study, the youngest participants were 12 years old, while two of the oldest individuals were approaching 19 years of age at the time of data collection. Like maxillary values, the variability was slightly greater in adolescents, as reflected by the standard deviation. The similarity in values may, in part, be attributed to the methodological approach used by the authors to assess molar inclination. In their CBCT-based studies, Yang and Chung reported mandibular first molar lingual inclination of 13.3° in adolescents and 13.1° in adults [12], and Alkhatib and Chung reported mandibular first molar lingual inclination of 12.6° in adults [1].

It should be noted that in Andrews’ six keys to normal occlusion, the third key refers to the crown inclination, which he measured from buccal crown surfaces, rather than the long axis of the crown. Thus, Andrews documented a 27° lingual crown inclination for maxillary molars and a 46° lingual crown inclination for mandibular molars [18]. In contrast, our study measured the long axis of the crown, finding a buccal crown inclination of about 9° in the maxillary molars and a lingual crown inclination of about 15° in the mandibular molars.

Of the 114 maxillary molars assessed in our study, 6 exhibited nearly upright inclinations. Consequently, 94.74% of maxillary molars in both adolescents and adults demonstrated positive buccal inclinations. The remaining 5.26% of molars showed near-upright positions, among which only one molar presented a negative inclination (−0.6°), observed in an adolescent subject. In a CBCT-based study, Alkhatib and Chung observed a buccal inclination of maxillary first molars in 90.7% of the measured teeth, with a 5° lingual inclination being the most significant deviation [1].

In his Six Elements philosophy, Andrews asserted that each crown must be inclined to ensure the occlusal surfaces interface and function optimally with the opposing arch [19]. Clinically, the buccolingual inclination of molars is critical for diagnosing the transverse relationship between the maxillary and mandibular arches. According to Dawson, the curve of Wilson suggests that the inward inclination of the occlusal table provides optimal resistance to masticatory forces and facilitates the chewing process [7]. Furthermore, Ishida and Soma reported that the force applied to molars during the terminal occlusal phase shows a buccal direction on the lower molars [20]. Regarding this, Lee et al. analyzed CBCT data from 54 untreated Caucasian adults to assess the relationship between the buccolingual inclination of mandibular first molars and their respective alveolar bones [21]. Their findings indicated that all subjects exhibited lingual inclination of both the mandibular molars and alveolar bones, with a significant correlation between them.

In terms of whether the buccolingual inclination of molars differs across facial skeletal patterns, Janson et al. reported similar inclinations regardless of facial type [22]. Yet, Tsunori et al. found that short-faced individuals exhibit greater lingual molar inclination in the mandible [23]. Kawamura reported that the buccolingual inclination of mandibular molars was associated with facial types characterized by ramus height, gonial angle, and mandibular plane angle [24]. Similarly, Masumoto et al. found that the subjects with a smaller gonial angle and mandibular plane angle had more vertically positioned molars [2]. In terms of sagittal skeletal pattern, Golshah et al. reported that the maxillary molars show more buccal inclination in Class I and III than in Class I patients, and the mandibular second molars in Class II show more lingual inclination than in Class I patients [25].

The primary advantage of our method is the elimination of the need for CBCT scans, thereby removing radiation exposure, which is especially crucial for pediatric patients who are more susceptible to radiation damage. This approach allows for multiple measurements of buccolingual inclination throughout treatment without safety concerns. Furthermore, the procedure is cost-effective, easy to perform, and time-efficient.

Caution should be exercised in cases of gingival overgrowth, as the inability to visualize the entire clinical crown may compromise measurements taken at the cervical base.

Our study has several limitations. First, the adolescent group is small (*n* = 16), limiting the statistical power; future studies should aim for a larger sample size. Secondly, the lack of CBCT measurements precludes a detailed comparison with our digital model data. Future research incorporating 3D radiographic data is warranted. Thirdly, this study lacks data on the relationship between facial skeletal patterns and the buccolingual inclination of molars on digital models; therefore, further research is warranted. Lastly, while our single-examiner design eliminates inter-examiner variability, it also means the measurement technique has not been validated across multiple operators. Future studies should include inter-examiner testing.

## 5. Conclusions

1. In adolescents and adults with near-normal occlusion, measured from 3D digital models, a mean buccal inclination of approximately 9° was seen in the maxillary first molars, and a mean lingual inclination of about 15° was seen in mandibular first molars.
2. Within the limitations of the present sample and measurement protocol, 3D digital models provided reproducible crown-based measurements of first molar buccolingual inclination, with values similar to those previously reported in CBCT-based studies. Direct validation against CBCT measurements in the same subjects and evaluation in patients with transverse discrepancies or post-treatment records are needed before broader diagnostic or outcome-related claims can be made.

## Figures and Tables

**Table 1:** The ICC and paired *t*-test results of each measurement.

Parameter	ICC with 95% CI	*p* Value of Paired *t*-Test
Right Maxillary Molar Inclination	0.96 [0.816, 0.993]	0.116
Left Maxillary Molar Inclination	0.99 [0.956, 0.998]	0.774
Right Mandibular Molar Inclination	0.94 [0.721, 0.987]	0.779
Left Mandibular Molar Inclination	0.96 [0.837, 0.993]	0.686

**Table 2:** Descriptive statistics for adolescent and adult maxillary first molar buccolingual inclination.

Parameter	Adolescent Maxillary First Molar	Adult Maxillary First Molar
Number of Subjects	16	41
Number of Molars	32	82
Mean	9.0	9.1
95% CI of Mean	[7.2, 10.9]	[8.1, 10.1]
Median	9.6	9.4
Minimum	−0.6	0.1
Maximum	20.0	19.5
Range	20.6	19.4
Standard Deviation (SD)	5.2	4.6
Standard Error of Mean (SEM)	0.9	0.5

**Table 3:** Mixed-effects analysis for adolescent vs. adult maxillary first molar inclinations.

Parameter	Result					
Fixed effect	Treatment (between columns)					
Random effect	Individual (between rows)					
*p*-value	0.7269					
F (DFn, DFd)	F (3, 70) = 0.4374					
Number of subjects	41 for the adult group, 16 for the adolescent group					
Tukey’s multiple comparisons tests	Adult R vs. Adult L	Adult R vs. Adolescent L	Adult R vs. Adolescent L	Adult L vs. Adolescent R	Adult L vs. Adolescent L	Adolescent R vs. Adolescent L
Predicted mean difference	−0.6244	−0.4826	0.6174	0.1418	1.242	1.100
90% CI of difference	−2.918 to 1.669	−3.697 to 2.732	−2.597 to 3.832	−3.072 to 3.356	−1.972 to 4.456	−2.571 to 4.771

**Table 4:** Descriptive statistics for adolescent and adult mandibular first molar buccolingual inclination.

Parameter	Adolescent Mandibular First Molar	Adult Mandibular First Molar
Number of Subjects	16	41
Number of Molars	32	82
Mean	−14.5	−15.6
95% CI of Mean	[−16.3, −12.7]	[−16.6, −14.6]
Median	−14.2	−15.8
Minimum	−24.1	−24.6
Maximum	−5.9	−2.3
Range	18.2	22.3
Standard Deviation (SD)	5.0	4.6
Standard Error of Mean (SEM)	0.9	0.5

**Table 5:** Mixed-effects analysis for adolescent vs. adult mandibular first molar inclinations.

Parameter	Result					
Fixed effect	Treatment (between columns)					
Random effect	Individual (between rows)					
*p*-value	0.3780					
F (DFn, DFd)	F (3, 70) = 1.045					
Number of subjects	41 for the adult group, 16 for the adolescent group					
Tukey’s multiple comparisons tests	Adult R vs. Adult L	Adult R vs. Adolescent L	Adult R vs. Adolescent L	Adult L vs. Adolescent R	Adult L vs. Adolescent L	Adolescent R vs. Adolescent L
Predicted mean difference	0.2585	−1.816	−0.7601	−2.075	−1.019	1.056
90% CI of difference	−2.031 to 2.548	−5.025 to 1.393	−3.969 to 2.449	−5.284 to 1.134	−4.228 to 2.190	−2.608 to 4.721

## Data Availability

The data presented in this study are available on request.
